# Evaluation of the Efficacy of Pasteurized Autograft and Intramedullary Vascularized Fibular Transfer for Osteosarcoma of the Femoral Diaphysis

**DOI:** 10.1111/os.12528

**Published:** 2019-10-29

**Authors:** Tang Liu, Lin Ling, Qing Zhang, Yong Liu, Xiaoning Guo

**Affiliations:** ^1^ State Key Laboratory of Powder Metallurgy Central South University Changsha Hunan China; ^2^ Department of Orthopaedics, The Second Xiangya Hospital Central South University Changsha Hunan China

**Keywords:** Autografts, Femur, diaphyses, Limb Salvage, Osteosarcoma

## Abstract

**Objective:**

To assess the treatment of osteosarcomas of the femoral diaphysis through wide en bloc excision and reconstruction of the defect by pasteurized autograft combined with vascularized fibular transfer.

**Methods:**

A single‐center, retrospective study was performed of 15 selected patients (six females, nine males) whose lesion in the middle diaphysis of the femur were treated by en bloc excision and reconstruction of the bone defect with recycled pasteurized autograft combined with vascularized fibular graft between January 2000 and December 2011. The primary diagnoses of the patients were osteosarcoma (15 patients), and one of these patients had a pathological fracture. The mean patient age at the time of surgery was 22.3 years (range, 10–40 years). All these cases of bone tumors were staged according to Ennekingʼs criteria with three stage IIA cases, and 12 stage IIB cases. The patients were examined clinically and radiologically every month during the first 6 months after surgery to exclude the evidence of infection and local recurrence, then at 3‐monthly intervals for 2 years and then at 6‐monthly intervals for life. Function was assessed using the Musculoskeletal Tumor Society Scoring system (MSTS).

**Results:**

At a mean follow‐up of 65.1 months (range, 31–131 months), all the patients had achieved bony union at the final follow‐up. The mean time to bone union of the proximal junctions of vascularized fibula was 8.7 months (range, 6.0–13.0 months) and that of the distal junctions was 9.2 months (range, 6.0–12.0 months). Mean union time of the proximal junctions of the pasteurized autogenous bone was 14.3 months (range, 10.0–25.0 months) and that of the distal junctions was 15.6 months (range, 10.0–27.0 months). There were two fractures of the pasteurized bone during the operation. One patient, in whom the plate had been removed after union at 3 years postoperatively, sustained a fracture in a fall. This was treated with external fixation and united uneventfully. One patient, in whom two of the proximal screws had been broken, developed coxa vara. There was no infection. There were three pulmonary metastases and no local recurrence. The mean function score was 81.8%. Five patients died of their underlying disease, and the disease‐specific survival of patients was 69.1%.

**Conclusions:**

Pasteurized autogenous bone graft combined with a vascularized fibula graft is a useful reconstruction method for large bone defects after resection of osteosarcoma in the femur.

## Introduction

Osteosarcoma is one of the most common primary bone tumors, which mainly affects children and adolescents. Current treatment for osteosarcoma involves surgical resection and multi‐agent neoadjuvant and adjuvant chemotherapy. This combined treatment has significantly improved the 5‐year survival rate. The introduction of chemotherapy combined with the development of imaging and surgical techniques have made it possible to perform bone tumor resections and limb‐sparing surgery in more than 80% of cases without incurring increased mortality^1^. Large bony defects resulting from surgical resection of malignant bone tumors present a difficult reconstructive challenge. Various methods are available to the reconstructive surgeon, such as allograft, endo‐prostheses, allograft–prosthetic composite (APC), pasteurized autograft, vascularized bone transplantation, and so on^1^, ^2^, ^3^. However, the optimal reconstruction procedure after wide resection of bone tumors is debatable.

Allograft reconstruction was championed in the 1970s as a biologic solution to the problem of restoring a segmental defect of the skeleton. Despite technical improvements in the method of fixation, and in the processing of the allograft to preserve cartilage cells and reduce contaminants, this method of reconstruction has significant complications. These include early complications, such as infection, nonunion and joint instability, and late complications such as instability and allograft fracture. Overall complication rates can exceed 50%, including an infection rate of 30%. Endoprosthetic reconstruction is a highly successful and durable method for the restoration of skeletal integrity and joint function. Use of a cemented stem provides immediate fixation, which allows for early mobilization and rehabilitation. However, endoprosthetic reconstruction is associated both with mechanical failures (e.g. stem fracture, erosion, and failure of polyethylene components) and non‐mechanical failures (e.g. infection and aseptic loosening). APC can be viewed as a transitional step between allografts and endoprostheses. APCs were thought to provide the benefits of a biologic reconstruction along with the immediate stability achieved by a cemented endoprosthesis. Experience has shown that this method has the same high rate of early complications (i.e. infection and nonunion) as does standard allograft reconstruction. Accordingly, this method is better suited for a patient undergoing revision of a failed allograft, rather than a patient undergoing chemotherapy for a sarcoma^1^, ^2^, ^3^.

We hypothesized that segmental tumor resection and endoprosthetic reconstruction would allow better local tumor control but result in worse function due to the reconstruction of the defect with recycled pasteurized autograft. Therefore, the purpose of this study was: (i) to assess the specific protocol for the treatment of osteosarcomas of the femoral diaphysis in a group of selected patients for limb salvage, with wide en bloc excision and reconstruction of the defect with recycled pasteurized autograft combined with vascularized fibular graft; (ii) to follow up the results of biological reconstruction in osteosarcoma patients after wide excision; and (iii) to analyze the oncological and functional differences between endoprosthetic reconstruction after wide resection and biological reconstruction after wide excision.

## Methods

### *Inclusion and Exclusion Criteria*

This was a single‐center, retrospective study conducted between January 2000 and December 2011 at our hospital. The inclusion criteria were as follows: (i) the primary diagnoses of the patients were osteosarcoma and the site of lesion in the middle diaphysis of the femur; (ii) patients were treated by en bloc excision and reconstruction of the bone defect with recycled pasteurized autograft combined with vascularized fibular graft; (iii) after an overview of our references, we compared our results with endoprosthetic reconstruction after wide resection cases in studies by Abudu *et al*. We picked out 13 cases from the study by Abudu *et al*. All these selected cases were patients with primary sarcomas of the femoral diaphysis; and (iv) the outcome measures are local recurrence, metastasis, bone union, revision, function measure (mean Musculoskeletal Tumor Society [MSTS] scoring system), and 5‐year survival rate.

Study exclusion criteria included: (i) the major neurovascular bundle cannot be free of tumor; (ii) the resection of the affected bone cannot leave a wide margin or a normal muscle cuff in all directions; and (iii) the patients who have undergone endoprosthetic reconstruction of the bone defect.

### *General Data*

A total of 15 selected patients (six females and nine males) were enrolled in this study, with one patient having a pathological fracture. The mean patient age at the time of surgery was 22.3 years (range, 10–40 years). All these cases of bone tumors were staged according to Ennekingʼs criteria with three stage IIA cases, and 12 stage IIB cases (Table 1).

**Table 1:** Patients’ data

Case names			Case 1	Case 2	Case 3	Case 4	Case 5	Case 6	Case 7	Case8	Case 9	Case 10	Case 11	Case 12	Case 13	Case 14	Case 15
Age(year)/gender			20/M	23/F	14/M	29/F	31/F	10/M	13/M	37/F	17/M	40/M	20/M	26/F	18/M	21/F	15/M
Stage			IIB	IIB	IIB	IIB	IIB	IIB	IIB	IIA	IIB	IIB	IIB	IIA	IIB	IIB	IIA
Specimen length (cm)			18.0	15.5	17.5	20.0	19.0	21.0	20.5	18.5	16.5	25.0	23.5	18.5	22.0	23.0	19.0
MSTS score (%)			80	83	81	85	82	71	77	80	90	85	80	65	89	87	92
Complications					Pulmonary metastasis			Shortening, coxa vara	Pulmonary metastasis		Accident	Osteoarthritis of knee	Fracture (Revision)	Heart failure due to chemotherapy		Pulmonary metastasis	
Union time (months)	Fibula Union	Proximal	7	8	10	7	6	8	12	7	9	11	10	8	7	13	7
		Distal	8	6	8	9	8	10	9	11	9	10	9	9	10	12	9.5
	Pasteurized Bone Union	Proximal	13	12	10	15	11	12	20	13	25	15	13	11	16	10	18
		Distal	12	15	13	11	12	10	27	12	18	17	18	15	23	17	14
Follow‐up time (months)			Alive, 131	Alive,105	Death, 39	Alive,89	Alive,81	Alive, 76	Death, 57	Alive,72	Death, 65	Alive, 56	Alive, 50	Death, 31	Alive,48	Death, 45	Alive, 31

### *Management Before Surgery*

Before operation, systematic examinations were performed to determine the degree of severity of the local disease and to distinguish the presence of metastasis including clinical assessment, X‐ray radiograph, SPECT scan, chest radiographs, and CT scan of lungs. Magnetic resonance imaging (MRI) was also performed to define the degree of the tumor, involvement of the soft tissues (particularly neurovascular bundle), and the level of bone resection. At diagnosis, there was no patient presenting lung metastases or skip metastasis. All patients with osteosarcoma received preoperative chemotherapies with a high dose of methotrexate, doxorubicin, ifosfamide, and cisplatin.

### *Operative Techniques*

All patients were anesthetized and in a supine position. Except in rare instances in which biopsy sites or soft tissue extension of tumor was prohibitive, a lateral femur approach was used and biopsy tracks, when present, were elliptically excised en bloc with the specimen. Wide resection was performed in all cases and all the bone defects occurred only in the diaphysis. To avoid intraosseous tumor extension, bone was resected 3.0 cm beyond abnormal uptake, as determined by preoperative MRI studies.

An ipsilateral vascularized fibula segment was taken with its periosteum and peroneal vessels. The length of the vascularized fibula exceeded the length of the resected bone defect by at least 4.0 cm. Microscopic anastomosis was achieved using the distal end of the femoral circumflex descending artery.

The bone was pasteurized in the following manner. After resection of the bone, soft tissue, gross tumor, and the intramedullary macroscopic portion of the tumor were cleared thoroughly from the specimen. It was then treated in saline, pre‐heated at 65°C for 45 min.

**Figure 1:**
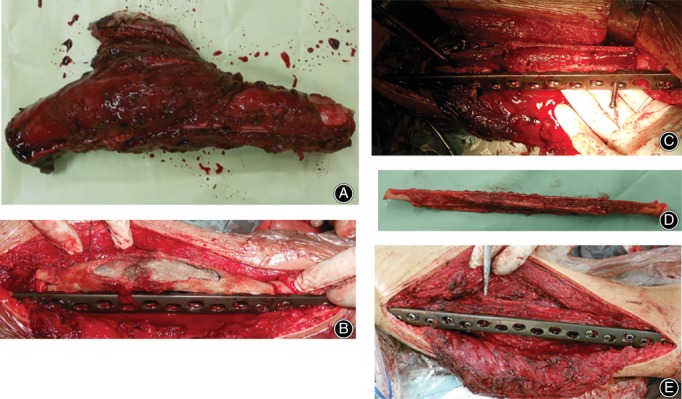
(A) Resected specimen intraoperatively. (B) Pasteurized autogenous bone was placed into the original anatomical site. (C) Pasteurized autogenous bone was fenestrated in order to intercalate the vascularized fibular. (D) Vascularized fibular was harvested. (E) Vascularized fibular was placed into the medullary canal of the pasteurized autogenous bone and fixed with a plate.

**Figure 2:**
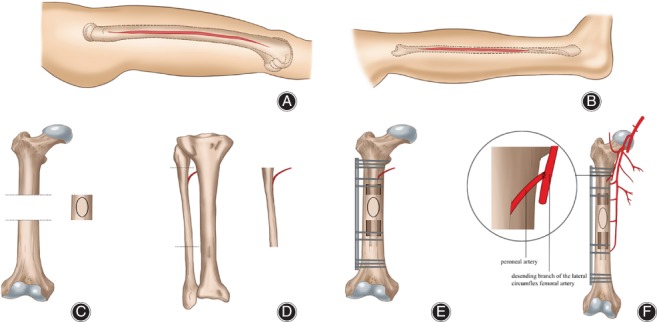
(A) Lateral femur approach was used to perform the en bloc excision with the tumor. (B) Lateral fibula approach was used to obtain the vascularized fibular. (C) Wide en bloc excision of the tumor. Then the specimen was pasteurized in saline, pre‐heated at 65°C for 45 min. (D) Vascularized fibular was harvested. (E) The pasteurized bone with vascularized fibular in its medullary canal was placed into the original anatomical site and fixed with a plate. (F) Microscopic anastomosis was performed.

The pasteurized bone was then reassembled at the original anatomical site. All patients had an intercalary autogenous bone graft. Fixation was accomplished as follows: the vascularized fibula was inserted into the pasteurized autogenous bone and host femur, and then fixed with a plate and screws (Fig. 1A‐E). Schematic diagrams of the surgical incisions and techniques are showed in Figs 2A‐F.

### *Management After Surgery*

The degree of tumor necrosis in response to the preoperative chemotherapy was rated according to the Huvos grading system^4^. Ten patients had grade IV response (complete tumor necrosis), two patients had grade III response (more than 90% necrosis), and three patients had grade II response (less than 90% necrosis). All the patients received postoperative chemotherapies except the three patients who had grade II response and were not compliant with the post‐operative chemotherapy regimen.

After operation, the reconstructed limbs were cast for 3 months, and a brace was adopted thereafter until radiographs showed union. Partial weight‐bearing was started when union of the vascularized fibula was noted, and weight‐bearing was done when union of the pasteurized autogenous bone was demonstrated on radiographs.

### *Outcome Measurement*

#### *Local Recurrence*

Local recurrence means the tumor has relapsed in the same place as the original tumor. The patients were examined clinically and radiologically every month during the first 6 months after surgery to exclude the evidence of infection and local recurrence, then at 3‐monthly intervals for 2 years, and then at 6‐monthly intervals for life.

#### *Metastasis*

Osteosarcoma that has spread from the initially affected bone to one or more sites in the body, distant from the site of origin, is called metastatic. The most common site to which osteosarcoma spreads is the lungs. A CT scan of the chest was performed every 3 months in the first year, and every 6 months thereafter. A bone scan was performed every 6 months in the first year and annually thereafter for life.

#### *Bone Union*

Bridging across three of four cortices in biplanar radiographs was considered evidence of consolidation.

#### *Revision*

Revision means surgery must be re‐done to replace a worn‐out joint replacement or amputation. The patients were examined clinically and radiologically to judge the evidence of infection, prosthesis loosening, tumor relapse, and so on.

#### *Function Measure*

Function was assessed using the Musculoskeletal Tumor Society Scoring system (MSTS)_2_.

### *Clinical Effect Evaluation*

After an overview of other studies, we compared our results with endoprosthetic reconstruction after wide resection cases from reports by Abudu *et al*. We selected 13 cases from the study by Abudu *et al*. (Table 2)^5^. All of these selected cases were patients with primary sarcomas of the femoral diaphysis. For evaluation of endoprosthetic and biological reconstruction methods, the two groups of patients were compared regarding their oncological, surgical, and functional outcome.

**Table 2:** Patients data from the study by Abudu *et al*

Case names	Case 1	Case 2	Case 3	Case 4	Case 5	Case 6	Case 7	Case 8	Case 9	Case 10	Case 11	Case 12	Case 13
Age(year)/gender	29/M	64/M	42/M	20/M	24/M	16/M	36/M	37/M	15/F	13/M	22/M	10/M	9/F
Specimen length (cm)	23	20	19	21	Unknown	18	23	23	28	18	24	23	16
MSTS score (%)	90	Unknown	70	Unknown	83	90	90	93	63	93	73	93	80
Complications	Loosening (Revision)	Local recurrence	Osteoarthritis of knee	Cement in the joint (Revision)	‐‐‐	Osteoarthritis of knee	‐‐‐‐	‐‐‐‐	Loosening (Revision)	‐‐‐‐	Loosening and shortening (Revision)	‐‐‐‐	Shortening
Follow‐up time (months)	Alive, 142	Death, 12	Alive, 72	Death, 32	Alive, 188	Alive, 82	Alive, 65	Alive, 6	Alive, 30	Alive, 66	Alive, 138	Alive, 30	Alive, 48

### *Ethics and Consent to Participate*

This study was approved by the Second Xiangya Hospital committee for clinical research (NO. 2012‐S231) and informed consent was obtained from the patients and the parents or guardians of the patients participating in the study.

### *Statistical Analysis*

SPSS v13.0 (SPSS™ Inc., Chicago, Illinois) was adopted to analyze statistics. Therapeutic variables (revision and function), pathological variables (Enneking stage, local recurrence, and metastatic disease) and demographic variables (gender, age, and duration of follow‐up) were examined. The endpoints of the study were local recurrence, progression of disease, and revision for any cause, such as infection, loosening, and so on. Descriptive summary statistics included means and ranges. Age and time intervals were regarded as continuous variables. All other covariates were modeled as categorical variables. Differences between means and proportions were tested with the Fisherʼs exact test or the Rank‐Sum test,and the Kaplan–Meier estimate was used to measure survival proportions. All tests were two‐sided and a *P*‐value <0.05 was considered significant.

## Results

### *General Results*

In the present study, the mean length of the femur defect after excision of the tumor segment was 19.8 cm (range, 15.5–25.0 cm). The mean length of surgical time was 309.2 min (range, 243–351 min). The mean blood loss during operation was 937.3 mL (range, 810–1251 mL). The mean wound drainage was 321.7 mL (range, 210–450 mL).

### *Assessment of Bone Union*

**Figure 3:**
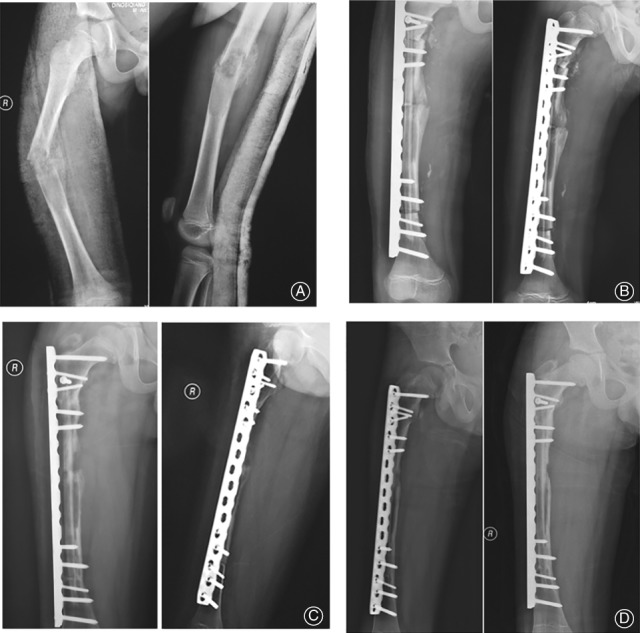
(A) The radiographs of a 10‐year‐old boy (case 6) with osteosarcoma of the right femur are shown. The femur was pathological fracture. (B) Radiographs taken 1 week postoperatively are shown. The length of the bone defect was 21 cm. (C) Radiographs taken 6 months postoperatively are shown. (D) Radiographs taken 12 months postoperatively are shown. Bone union and incorporation were achieved.

**Figure 4:**
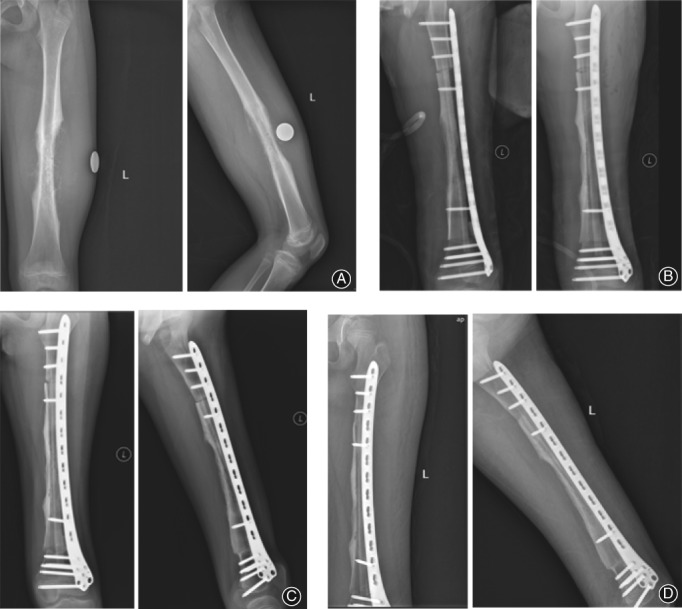
(A) The radiographs of a 13‐year‐old boy (case 7) with osteosarcoma of the left femur are shown. (B) Radiographs taken 1 day postoperatively are shown. (C) Radiographs taken 12 months postoperatively are shown. (D) Radiographs taken 27 months postoperatively are shown. Bone union and incorporation were achieved.

At a mean follow‐up of 65.1 months (range, 31–131 months), all the patients had achieved bony union at the final follow‐up. The mean time to bone union of the proximal junctions of vascularized fibula was 8.7 months (range, 6.0–13.0 months) and that of the distal junctions was 9.2 months (range, 6.0–12.0 months). The mean union time of the proximal junctions of the pasteurized autogenous bone was 14.3 months (range, 10.0–25.0 months) and that of the distal junctions was 15.6 months (range, 10.0–27.0 months) (Figs 3A‐D and 4A‐D).

### *Assessment of Complications*

There were two fractures of the pasteurized bone during the operation. One patient, in whom the plate had been removed after union at 3 years post‐operatively, sustained a fracture in a fall. This was treated with external fixation and united uneventfully. One patient, in whom two of the proximal screws had been broken, developed coxa vara. There was no infection.

Pulmonary metastasis occurred in three patients. Those patientsʼ pulmonary metastasis occurred at the time of 39 months, 57 months, and 45 months after surgery, respectively. One patient received revision at the time of 47 months postoperatively. Five patients died of their underlying disease. There was no local recurrence.

### *Clinical Effect Evaluation Outcomes*

We compared our results with endoprosthetic reconstruction after wide resection cases from reports by Abudu *et al*. There were no significant differences between the patients in our study and the study by Abudu *et al*. in age at surgery, gender, or tumor location.

#### *Metastasis*

**Figure 5:**
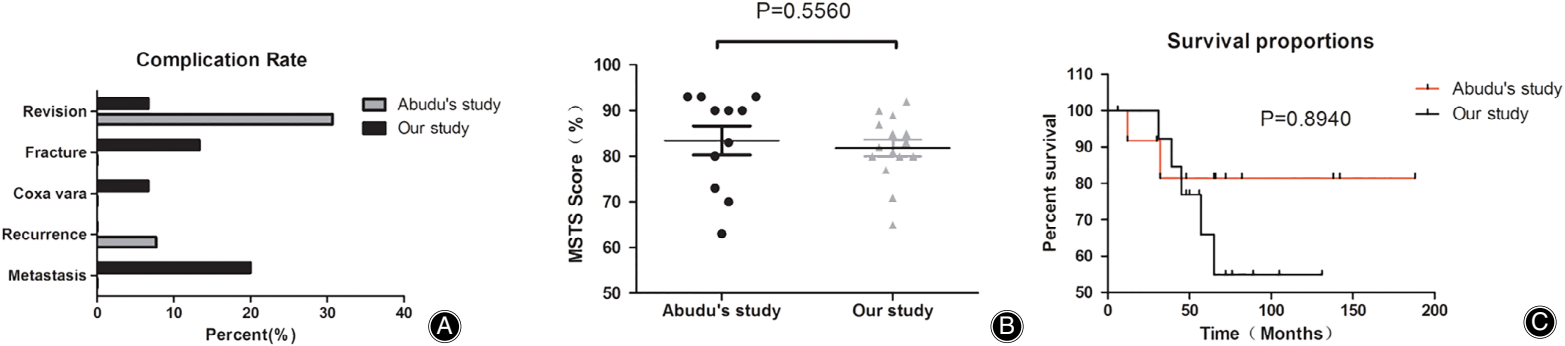
(A) Complication diagram showed that revision was much higher in the study by Abudu *et al*. (B) Functional evaluation showed there were no statistical differences between the two groups. (C) Survival proportions in study by Abudu *et al*. and our study.

Pulmonary metastasis occurred in three patients (20.0%) in our study while no pulmonary metastasis occurred in the patients in the study by Abudu *et al*. (Table 3 and Fig. 5A).

**Table 3:** Main patient and outcome characteristics comparison

Group names	Abudu *et al*. study	Our study	*P*‐value
Total	13	15	–
Mean age (years)	25.9 ± 15.6	22.3 ± 8.8	0.7860*
Sex	11M / 2F	9M / 6F	0.2210†
Specimen Length (cm)	21.3 ± 3.3	19.8 ± 2.7	0.4960*
Mean follow‐up (months)	70.1 ± 55.2	65.1 ± 28.0	0.7510*
Local recurrence	1	0	0.4640†
Metastasis	0	3	0.2260†
Mean MSTS	83.5 ± 10.6	81.8 ± 7.1	0.5560*
Revision	4	1	0.1530†
Five‐year survival rate	74.1%	69.1%	0.8940†

* Rank‐Sum test.

† Fisherʼs exact test.

#### *Local Recurrence*

There was no local recurrence in our study, while local recurrence occurred in one patient (7.69%) in the study by Abudu *et al*. (Table 3 and Fig. 5A). In our study, there was one revision (6.7%), while revisions were indicated in four patients (30.8%) in the study by Abudu *et al*. (Table 3 and Fig. 5A).

#### *Function Measure*

All patients were functionally assessed at the latest follow‐up. The mean MSTS_2_ score was 81.8% in our study and 83.5% in the study by Abudu *et al*. (Table 3 and Fig. 5B).

#### *Five‐Year Survival Rate*

In our study, five patients died of their underlying disease while all other patients were alive without evidence of disease at latest follow‐up. Consequently, the disease‐specific survival of patients in the present study was 69.1% at 5 years, where it was 74.1% in the study by Abudu *et al*.(Fig. 5C).We were under‐powered to detect statistically significant differences between groups, and thus lack of a significant finding should not be intercepted as no difference. Some of the differences that failed to reach statistical significance, such as the revision rate (7% in our study *vs*. 30.8% in the Abudu *et al*. study), was large^5^.

## Discussion

In this study, we used a pasteurized intercalary autogenous bone graft combined with a vascularized fibula graft for bony defects after tumor resection in the patients with osteosarcoma and acquired good oncological and functional outcomes. Furthermore, we were able to prove that prosthetic reconstruction was similar to reconstruction with recycled pasteurized autograft in terms of local recurrence and metastasis, while a higher rate of revision was noted in patients with prosthetic reconstruction.

### *Limitation of the Study*

We note several limitations in this study. The study was retrospective, and the number of patients was relatively small. Maybe it related to the fact that there wasn't any statistically significant difference between our study and that of Abudu *et al* 5. Although only three patients developed pulmonary metastases and none developed local recurrence, longer follow‐up is needed as these were malignant lesions with a possibility of late recurrence.

### *Complication Rates*

High complication rates were reported in the use of recycled pasteurized autograft without a vascularized fibular graft. Manabe *et al*. described a complication rate of 52% in 25 patients who had reconstruction surgery with pasteurized autogenous bone^6^. In their study, fracture (12%) and infection (20%) were the main complications. Ahmed *et al*. reported 22 sarcoma patients whose bone tumor excision followed by pasteurization and reimplantation^7^. In their study, fracture (13.6%), graft collapse (9.0%), and infection (9.0%) were the major complications encountered. Sugiura *et al*. described a series of 19 patients who received only pasteurized autogenous bone^8^. Infection was noted in one patient, fracture was present in two patients, and pseudoarthrosis was found in six patients. In their study, the complication rate was nearly 50%. In a previous study, we treated malignant bone tumors of the distal tibiae with en bloc intra‐articular excision and ankle arthrodesis using recycled pasteurized autograft; infection occurred in two patients (20.0%) while the nonunion rate was 54.5%^9^. Manfrini *et al*. reported on 24 patients with bone tumors that had intercalary segments of tibia or femur reconstructed with the fibula inside the massive allograft^10^. In their study, the rate of fracture or nonunion of allograft was 33.3%. Li *et al*. reported 11 patients that had intercalary resection of lower extremity malignancy underwent reconstruction with an allograft and vascularized fibular construct^11^. There were no allograft fractures or infections. Nonunion was noted in one patient (9.1%). In the current study, bone defects were reconstructed by the combination of vascularized fibula and pasteurized autogenous bone grafts. All the patients had achieved bony union without infection. There were two fractures of the pasteurized bone during the operation and one patient sustained a fracture in a fall 3 years post‐operatively.

Chemotherapy has been reported to increase the infection and decrease bony modeling in a dose‐dependent manner. A series of 112 patients treated with massive bone allografts for osteosarcoma was reported^12^. The study showed the incidence of delayed union was 49% and identified that the incidence of delayed union was significantly increased by the use of chemotherapy^13^. Another study also indicated that the incidence of the nonunion was significantly increased in the setting of chemotherapy^14^. Li *et al*. reported that the time of osseous union in the patients who had chemotherapy was longer than that in patients who did not receive chemotherapy. In our series, all the patients received chemotherapy^11^. Mean union time of the proximal junctions of the pasteurized autogenous bone was 14.6 months and that of the distal junctions was 13.3 months, which was comparable with that in previous studies, with ranges between 6.1 months and 25.0 months^11^, ^15^, ^16^. We had no cases of bony infection, which is a significant improvement in comparison to previous studies^15^, ^16^. The lower infection rate may be attributed to better vascular circumstance provided by the grafted vascularized fibula.

### *Functional Evaluation*

The MSTS score is commonly used for functional evaluation after grafting. Several studies have been done using this evaluation system. Manabe *et al*. reported an MSTS score of 86% in the lower limbs in 25 patients who had autogenous pasteurized bone grafting^8^. Suk *et al*. reported an MSTS score of 77% in the lower limbs in 12 patients who had autogenous pasteurized bone grafts^17^. Sugiura *et al*. reviewed pasteurized intercalary autogenous bone graft combined with a vascularized fibula graft in 15 patients with malignant bone tumors^15^. The mean total postoperative functional evaluation score was 81%, and the mean MSTS score for 12 patients with bone defects at sites from the diaphysis to the metaphysis was 78%. In the present study, the mean post‐operative functional score was 80.8%, which was comparable to others.

### *Fibula Graft Outcome*

As described by Sugiura *et al*., plate fixation is technically difficult in bone defects of 15 cm or larger^15^. For such cases, they fixed the fibula outside the pasteurized autogenous bone and used an intramedullary nail inside the marrow, while in our study all the patients adopted plate fixation and achieved good outcomes.

The study by Manfrini *et al*. suggests that free fibular flaps will incorporate into the allograft^10^. Li *et al*. observed that abundant callus originated from the outerlayer of the fibula and united the fibula with the host bone and allograft^18^. They also found that even if contact between the allograft and host bone is less than optimal, vascularized bone flap facilitates the host–allograft union at the level of the osteotomy. Pasteurized autogenous bones are similar to allografts, in that both possess bone induction ability and bone conductive ability^8^. Sugiura *et al*. reviewed pasteurized intercalary autogenous bone graft combined with a vascularized fibula graft and stated that the addition of a vascularized fibula graft seems to promote the theoretically anticipated remodeling process and more complete bone was contained than would be expected without the graft^14^. Our results indicate that pasteurized intercalary autogenous bone graft combined with a vascularized fibula graft is a useful reconstruction method for large bone defects after resection of osteosarcoma in the femurs.

Therefore, we make a conclusion that pasteurized autogenous bone graft combined with a vascularized fibula graft is a useful reconstruction method for large bone defects after resection of osteosarcoma in the femurs.

## Author contributions

Q. Z., T.L., L. L., and X. G. conceived the study and designed the study. Y. L., T.L., L. L., and X. G. provided the study materials. L. L., T.L. and X.G. performed the data analysis. All authors contributed to the interpretation and discussion of the results and wrote the manuscript. All authors have approved the manuscript for submission.
